# Spontaneous Tetanus

**Published:** 1831

**Authors:** 


Spontaneous Tetanus.

[Hôpital St. Antoine.]

*Case.* Margaret P. aged 56, of good constitution, was suddenly seized, without any apparent cause, with acute pain in the lumbar region and all along the back; and shortly afterwards with strong and general rigidity, being deprived of motion in the lower extremities. On the 5th day from the commencement of the attack, (viz. 8th April, 1831,) she entered the above-mentioned hospital, presenting the following symptoms—general contraction—fore-arms half bent—total inability to stand—jaws firmly closed—speaks with difficulty—intellects unaffected—face flushed—eyes watery—deglutition difficult—respiration rather laborious—pulse full and hard. Bled to 12 ozs.—diluent drink. 6th. The blood was buffed — other symptoms much the same. She was cupped on the loins—took opiates internally, and had opiate frictions externally employed. In the evening the breathing became very laborious, and all the symptoms were exasperated. She died the same evening in strong convulsions.

*Dissection.* The meninges of the brain were much injected, but there was nothing else remarkable in the brain, except that more red points than usual were observable on slicing it. There was a great quantity of serous fluid in the theca vertebralis, and the membranes enveloping the spinal marrow were of a rose colour, with sanguineous arborisations over the surface of the medulla itself. The anterior columns were evidently softened, and made no resistance to the scalpel. On close examination, in fact, it was found reduced to a jelly, especially in the lumbar region, and also in the cervical. The posterior columns were of the usual and healthy consistence. The origins of the nerves, anterior and posterior, presented nothing unusual—nor did the pneumogastric nerves, the semilunar ganglion, or the great sympathetic nerves.—*Rev. Med.*

